# Interfacially Assembled Hollow AuAg Nanostars on Flexible PDMS for Sensitive SERS Detection

**DOI:** 10.3390/molecules31193561

**Published:** 2026-10-07

**Authors:** Jiatong Li, Meiyu Liu, Xinyuan Meng, Jiayi Hou, Chaojie Xu, Wenshi Zhao, Jie Zhao

**Affiliations:** 1 Key Laboratory of Bionic Engineering, Ministry of Education, Jilin University, Changchun 130022, China; lijt1824@mails.jlu.edu.cn (J.L.); liumeiyu1234562025@163.com (M.L.); jyhou25@mails.jlu.edu.cn (J.H.); xucj25@mails.jlu.edu.con (C.X.); 2 Key Laboratory of Functional Materials Physics and Chemistry of the Ministry of Education, Jilin Normal University, Changchun 130103, China; 18943031115@163.com

**Keywords:** surface-enhanced Raman scattering, PDMS-AuAg alloy nanostars, finite-difference time domain, methylene blue

## Abstract

Optimizing the morphology and structure of noble metal nanomaterials is critical for the design of high-performance surface-enhanced Raman scattering (SERS) substrates for trace analyte detection. A plasmon-tunable SERS platform consisting of AuAg alloy nanostars anchored on a hydrophobic polydimethylsiloxane (PDMS) matrix was developed, which combined the chemical stability of Au and the strong localized surface plasmon resonance of Ag. AuAg alloy nanostars with sharp tips were synthesized via a galvanic replacement reaction, in which the precise modulation of Ag seed concentration enabled a morphological evolution from solid to hollow nanostructures. The optimized AuAg-3 nanostars exhibited the highest SERS response due to a dual synergistic mechanism: strong electromagnetic enhancement at the sharp branched tips and additional plasmonic enhancement from the hollow cavity through light scattering and trapping, which increased the optical path length and excitation efficiency. Furthermore, these nanostars were transferred onto a flexible PDMS film through interfacial self-assembly. The resulting hydrophobic PDMS-AuAg-3 substrate synergistically combined the strong electromagnetic enhancement from the nanostars with efficient analyte enrichment driven by the hydrophobic surface, achieving an ultra-low detection limit of 2.4 × 10^−10^ M for methylene blue (MB). This strategy effectively balances high sensitivity and excellent reproducibility, offering a robust pathway for the development of next-generation flexible SERS sensors.

## 1. Introduction

The precise identification and quantitative detection of textile dyes in complex real-world matrices are crucial for product safety and environmental regulation. Surface-enhanced Raman scattering (SERS), a powerful molecular fingerprinting spectroscopic technique, has demonstrated immense potential in trace-level analysis due to its ultra-high sensitivity [1,2,3,4]. Nevertheless, the practical application of SERS, particularly for monitoring environmental pollutants, still faces significant challenges [5,6]. While SERS substrates are fundamental to signal amplification [7], conventional colloidal substrates composed of Au or Ag nanoparticles often exhibit poor signal reproducibility caused by the random and uneven distribution of electromagnetic hotspots [8,9]. Furthermore, their limited capacity to enrich target molecules from the sample matrix restricts the ultimate detection limits [10]. These inherent drawbacks greatly limit the reliability of SERS quantitative analysis and hinder its practical application in real-sample analysis.

To overcome the above challenges, significant research efforts have been directed toward designing novel noble metal nanostructures with precise composition and morphology [11,12,13]. Noble metal nanoparticles are crucial for generating localized surface plasmon resonance (LSPR), which enhances the electromagnetic field and, consequently, the Raman signal of analyte molecules [14]. Although Au nanostructures exhibit tunable LSPR and superior chemical stability [15], their SERS enhancement factor is generally lower than that of Ag [16,17]. Conversely, while Ag nanoparticles provide superior LSPR effects, they are susceptible to oxidation under environmental conditions [13]. Studies suggest that alloying Au with Ag can improve stability while maintaining excellent LSPR performance [18,19]. In addition, nanoparticles featuring sharp edges or intrinsic nanogaps can further intensify the local electric field [20]. Specifically, anisotropic star-shaped nanoparticles demonstrate superior SERS enhancement efficiency due to their sharp tips, while nanogaps between adjacent nanostructures further promote plasmonic coupling [21]. Furthermore, the LSPR characteristics can be finely tuned by modulating the Au/Ag ratio to achieve optimal matching with specific laser excitation wavelengths [22,23]. Therefore, plasmon-tunable AuAg alloy nanostars are considered highly promising candidates for SERS substrates, offering a platform for high-intensity and strong electromagnetic enhancement.

To achieve ultra-sensitive sensing, high-performance SERS substrates must be integrated with sample enrichment capabilities [24,25]. In recent years, polydimethylsiloxane (PDMS) has garnered significant attention in SERS applications, owing to its exceptional transparency in the visible region and inherent hydrophobicity [26,27]. The hydrophobic nature of PDMS facilitates the spontaneous pre-concentration of pollutant molecules, effectively increasing the molecular concentration on surfaces of SERS substrates [28]. More importantly, the high flexibility of PDMS allows for the modulation of SERS hotspot density through simple mechanical deformation, ensuring excellent portability [29,30]. However, the current fabrication methods for hydrophobic SERS substrates often rely on costly techniques such as photolithography, which limits their widespread practical application [31]. Meanwhile, the relationship between the morphology, particularly hollow architecture and branch characteristics, of AuAg nanostars and their SERS performance remains insufficiently understood [32]. Therefore, developing high-performance, hydrophobic and LSPR-tunable SERS substrates through simple processing methods is imperative for ultra-sensitive detection of pollutants.

In this study, we synthesized plasmon-tunable AuAg alloy nanostars and investigated their morphology evolution and underlying formation mechanism through combined compositional and structural analyses. Subsequently, methylene blue (MB) was used as the Raman probe molecule to evaluate the SERS performance of AuAg alloy nanostars with different morphologies. Meanwhile, finite difference time domain (FDTD) stimulations were performed to correlate morphology-dependent SERS activity with local electromagnetic-field distributions. In addition, PDMS-AuAg nanostar substrates exhibiting superior SERS performance were successfully fabricated via interfacial self-assembly and subsequent transfer onto flexible PDMS films, achieving a detection limit as low as 2.4 × 10^−10^ M. PDMS could confine the target molecules within a localized area, thereby increasing the local analyte concentration and enhancing detection sensitivity. We envision that this PDMS-AuAg alloy nanostars SERS platform will offer potential for sensitive detection of trace environmental pollutants.

## 2. Results and Discussion

### 2.1. Formation Mechanism and Structural Characterization of AuAg Alloy Nanostars

**Figure 1:**
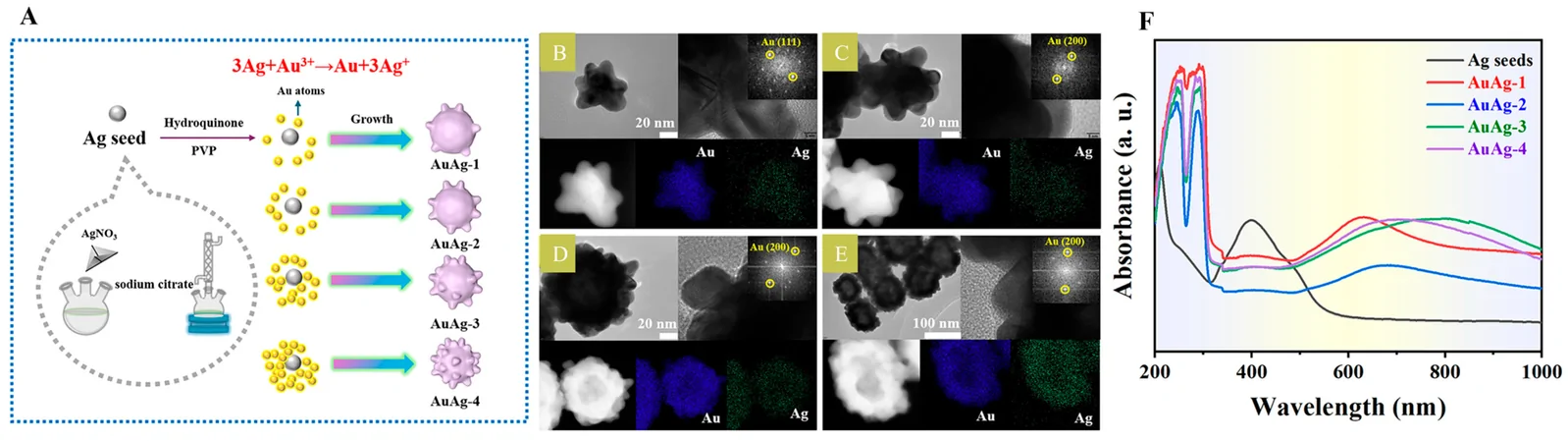
(**A**) Schematic diagram of the synthesis of AuAg alloy nanostars with different volumes of Ag seeds (1, 2, 3 and 4 mL); TEM, HRTEM, high-angle annular dark-field scanning transmission electron microscopy (HAADF-STEM) images, and corresponding element mapping images (Au and Ag) of (**B**) AuAg-1, (**C**) AuAg-2, (**D**) AuAg-3 and (**E**) AuAg-4; (**F**) UV–Vis spectra of Ag seeds, AuAg-1, AuAg-2, AuAg-3 and AuAg-4.

Figure 1A shows the schematic illustration of the synthesis procedures for AuAg alloy nanostars with different volumes of Ag seeds (1, 2, 3 and 4 mL). Firstly, Ag seeds with an average diameter of 40 nm were prepared (TEM images shown in Figure S1). Subsequently, AuAg alloy nanostars were formed through the galvanic replacement reaction between Ag seeds and HAuCl_4_·3H_2_O, employing hydroquinone and PVP as the reducing agents and surfactants, respectively. In this process, Ag seeds serve as sacrificial templates because Ag possesses a lower reduction potential than Au and is oxidized by Au^3+^ ions with a higher reduction potential [33]. Specifically, owing to the higher standard reduction potential of Au^3+^/Au^0^ compared to Ag^+^/Ag^0^, Ag seeds are oxidized and dissolved, while Au^3+^ ions are reduced and deposited onto the surfaces of the template [34]. Furthermore, hydroquinone selectively adsorbs onto specific crystal planes during Au reduction, inhibiting their growth; this anisotropic growth is the primary driver for the formation of the branched structures [35].

Energy-dispersive X-ray spectroscopy (EDS) analysis (Figure S2) was employed to quantify the atomic percentages of Au and Ag in the AuAg alloy nanostars. Table 1 confirms the coexistence of Au and Ag elements in all samples [36]. The influence of different additional amounts of Ag seeds on the final morphology of AuAg alloy nanostars was then investigated. It can be seen from Figure 1B–E that the amount of Ag seeds added governs the structural evolution from solid to hollow cores and modulates the branch density. Figure 1B shows that AuAg-1 exhibits a solid nanostar morphology with an average diameter of 60 nm and sparse branches approximately 20 nm in length. Elemental mapping image confirms the homogenous distribution of Au and Ag, indicating the successful synthesis of hybrid alloy. As the added amount of Ag seed solution increases from 1 mL to 2 mL (Figure 1C, AuAg-2), the particle size increases to 80 nm, the branch length decreases to 15 nm, and the branch density significantly increases. This suggests that a higher concentration of Ag seeds provides more nucleation sites for the limited supply of Au atoms, resulting in more numerous but shorter and less sharp branches [37].

Notably, increasing the Ag seed volume from 2 mL to 4 mL induced a gradual transition from solid to hollow nanostructures, as presented in Figure 1D,E. AuAg-3 shows a distinct hollow structure with an average particle diameter of approximately 110 nm and a hollow cavity diameter of about 36 nm. AuAg-4 displays a more pronounced hollow architecture with an average particle diameter of approximately 126 nm and a hollow cavity diameter of about 54 nm. This phenomenon can be attributed to the altered kinetics of the galvanic replacement reaction caused by changes in the Au^3+^/Ag ratio. Initially, Au atoms deposit on the surfaces of Ag seeds, acting as electron acceptors [38]. At a high Au^3+^/Ag ratio, the rapid deposition of a dense Au shell passivates the Ag core, preventing outward diffusion and resulting in solid structures. Conversely, at higher Ag seed concentrations (lower Au^3+^/Ag ratios), Au grows epitaxially on the initial layer. However, the oxidation and dissolution of the Ag core proceed, and the vacancies left by Ag atoms are not fully replenished by Au, leading to the formation of interior voids. It can also be observed from TEM element mapping images in Figure 1D,E that Ag signals are mainly localized at the outer regions of the nanoparticles. Meanwhile, the presence of residual Ag element inside corroborates that Ag seeds are not completely etched, thereby strongly supporting the above explanation. High-resolution TEM (HRTEM) observations of the evolution of lattice fringes from (111) to (200) crystal planes provide additional evidence for the structural reconstruction during the growth of AuAg alloy nanostars [39].

The intrinsic LSPR properties of noble metal nanoparticles are critical for maximizing SERS detection performance. The effect of different Ag seed concentrations on the UV–Vis absorption spectra of AuAg alloy nanostars was investigated. It should be noted that the UV–Vis spectra shown in Figure 1F were recorded after diluting the original dispersions with different amounts of water to obtain absorbance values within the instrument’s linear range. The absorption spectrum of Ag seeds exhibits a typical LSPR peak at 400 nm. In contrast, AuAg-1 displays a broadened LSPR peak significantly redshifted to 630 nm. This redshift is attributed to the formation of anisotropic branched structures with sharp tips, which induce enhanced plasmon coupling and contribute to the tunable optical response of AuAg nanostars [40,41]. As the added amount of Ag seeds increases from 1 to 3 mL, the LSPR peak in the absorption spectrum undergoes further redshift because of the increased branch density. However, when the amount of Ag seeds increases to 4 mL, the LSPR peak of AuAg-4 is blueshifted. This reversal likely occurs because excessive Ag content causes the optical properties of AuAg alloy nanostars to shift more towards the intrinsic characteristics of Ag seeds.

### 2.2. SERS Sensing Performance of AuAg Alloy Nanostars

**Figure 2:**
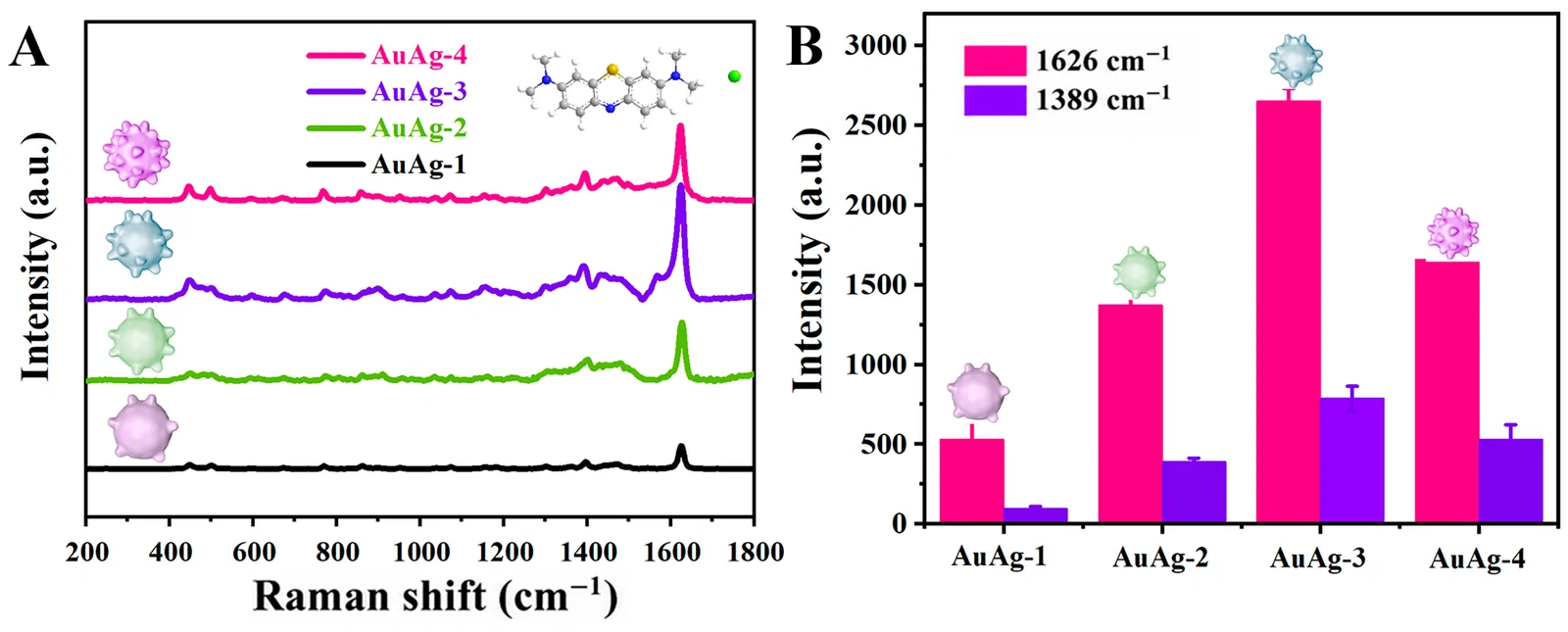
(**A**) SERS spectra and (**B**) SERS intensities of MB (10^−7^ M) at 1389 and 1626 cm^−1^ adsorbed on AuAg-1, AuAg-2, AuAg-3 and AuAg-4. The error bars represent the standard deviations from three measurements.

To evaluate the intrinsic SERS activity of the AuAg nanostars, measurements were carried out by choosing MB as the Raman reporter molecule (Raman spectrum of MB displayed in Figure S3). SERS spectra of MB adsorbed on AuAg-1, AuAg-2, AuAg-3 and AuAg-4 are depicted in Figure 2A, while Figure 2B presents the intensities at characteristic peaks of 1389 cm^−1^ (symmetric C-N stretching) and 1626 cm^−1^ (C-C ring stretching) [22,42]. All the samples exhibit strong SERS enhancement, which is mainly due to the following two reasons. On one hand, the sharp branches and interstitial nanogaps of the AuAg alloy nanostars enable analyte molecules to be trapped near the metal surface, creating abundant plasmonic coupling sites and electromagnetic hotspots. This synergistic effect is the foundation of the excellent SERS enhancement ability exhibited by the AuAg alloy nanostars. On the other hand, nanostars with multiple anisotropic branches can form a large number of SERS hotspots at the tips, thereby enhancing SERS performance [43,44]. Meanwhile, it can be observed that the SERS intensity is closely related to the added amount of Ag seeds. Compared to AuAg-1 and AuAg-2, AuAg-3 exhibits the highest SERS enhancement. This superior performance is attributed to the cavity-mediated plasmonic enhancement driven by multiple internal reflections within the hollow architecture [45]. However, when the added amount of Ag seeds further increases to 4 mL, the SERS intensity decreases. The abnormal decrease in SERS performance is due to the excessive addition of Ag seeds, which leads to the morphological transformation of the initially sharp nanostars into more rounded structures with fewer sharp tips, thereby reducing the number of effective hotspots. The observation is consistent with the corresponding TEM observations in Figure 1E. It is evident that the SERS enhancement of MB is closely related to the morphology of AuAg alloy nanostars. Regarding the calculated enhancement factor (EF), see the Supplementary Materials for details.

**Figure 3:**
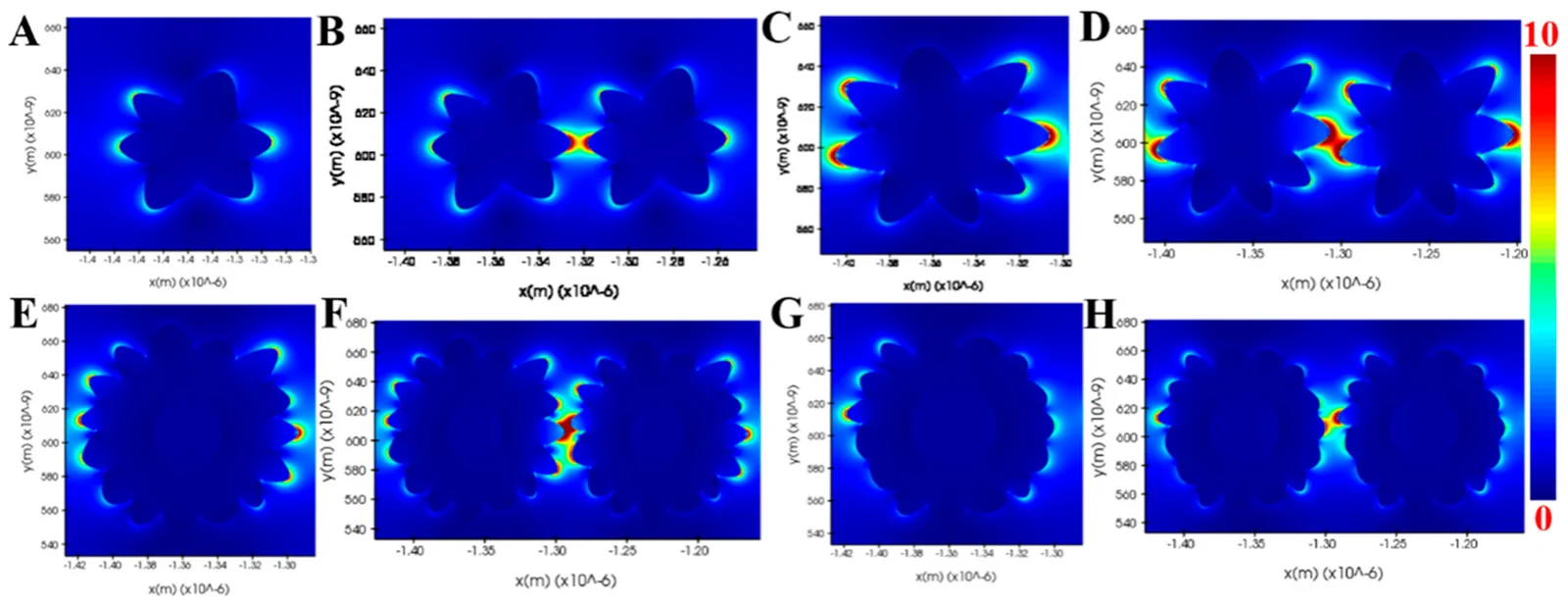
Simulated electric field distributions of individual and adjacent (**A**,**B**) AuAg-1, (**C**,**D**) AuAg-2, (**E**,**F**) AuAg-3 and (**G**,**H**) AuAg-4.

To reveal the influence of morphology on the electromagnetic enhancement effect, FDTD simulations were performed to calculate the local electric field distributions of different microstructures of AuAg alloy nanostars. It can be seen from Figure 3 that SERS hotspots are concentrated at the branch tips and in the interstitial regions between adjacent nanostars [23]. The high charge density at the sharp branches results in intense local electromagnetic fields [46]. As shown in Figure 3A–H, with the increase in Ag seeds, the electric field intensity of AuAg alloy nanostars significantly increase. Among them, the electric field intensity of AuAg-3 is the highest, which is consistent with the trend of SERS enhancement in Figure 2. In this state, the tip enhancement effect of the branch tip overlaps with the gap plasmon resonance inside the cavity, further enhancing the efficiency of electromagnetic field localization. Therefore, the excellent performance of AuAg-3 is attributed to its unique hollow structure, which triggers a dual synergistic mechanism combining hollow-structure-associated plasmonic enhancement and tip-induced field localization. In contrast, AuAg-4 shows a decreased electric field intensity, which can be attributed to the rounding of the sharp tips, resulting in fewer effective hotspots. The above results indicate that the morphology-controlled local field enhancement is the dominant factor governing SERS activity. On the basis of its optimal electromagnetic enhancement, AuAg-3 was chosen as the SERS substrate for subsequent experiments.

### 2.3. SERS Sensing Performance of PDMS-AuAg-3 Flexible Substrate

**Figure 4:**
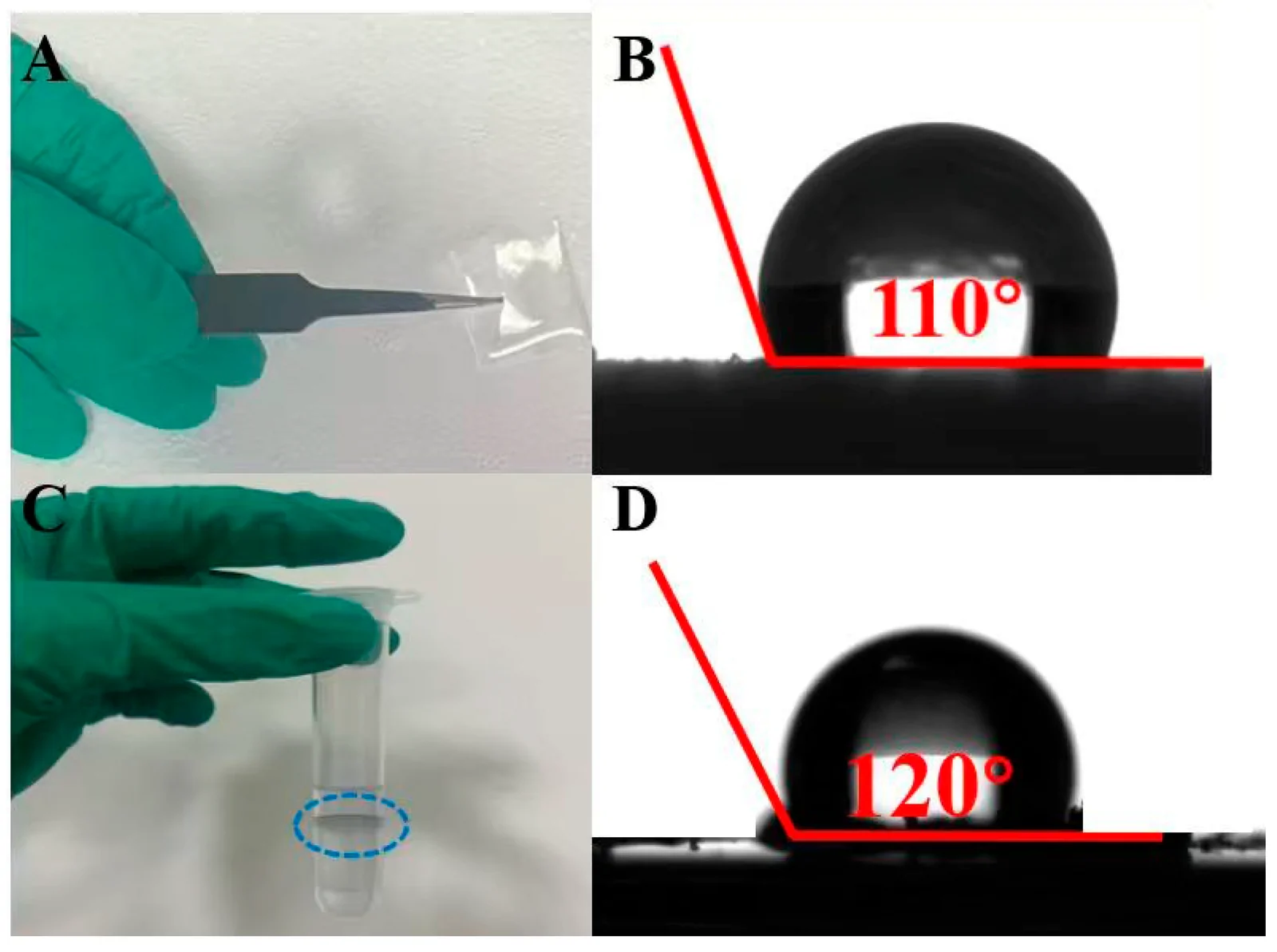
(**A**) Optical photograph and (**B**) water contact angle of PDMS film; (**C**) optical photograph and (**D**) water contact angle of PDMS-AuAg-3 flexible SERS substrate.

To address the issues of nanoparticle aggregation and uneven hotspot distribution encountered by the AuAg-3 substrate in practical SERS detection, integrating AuAg-3 substrate into a flexible PDMS substrate is a key strategy to improve the stability and practical applicability of SERS detection [47]. The core of the preparation of this flexible substrate lies in the use of oil–water–oil three-phase interface self-assembly technology, which can achieve precise transfer and single-layer arrangement of hollow AuAg nanostars from the liquid phase to the solid phase interface [48]. Specifically, by introducing CH_2_Cl_2_ and hexane to create a specific oil–water interface environment, the hollow AuAg nanostars spontaneously form dense and uniform monolayer films driven by interfacial tension, effectively avoiding random stacking and the coffee-ring effect typically associated with traditional drop-coating methods. The assembled nanostar monolayer was subsequently transferred onto cured PDMS films, forming the PDMS-AuAg-3 flexible composite substrate. Figure 4A demonstrates the high transparency of the PDMS film. This optical transparency provides favorable conditions for SERS measurements by facilitating the propagation of excitation and Raman scattered light. Contact angle measurements (Figure 4B) reveal a contact angle of 110°, confirming the inherent hydrophobicity of PDMS. After transfer of the nanostar film, the contact angle between a water droplet and the PDMS-AuAg-3 surface increases to 120°, indicating further enhanced surface hydrophobicity (Figure 4C,D). This change in wettability not only promotes the enrichment of analyte molecules towards hotspot regions during evaporation, but also effectively prevents the aggregation between adjacent nanostars, thereby increasing the number of hotspots. To directly verify the inhibitory effect of the coffee-ring effect on hydrophobic PDMS, optical microscope images of dried MB droplets were collected on PDMS and hydrophilic silicon wafers. In addition, the flexible nature of PDMS substrate endows it with mechanical flexibility and conformability, enabling in situ detection on irregular sample surfaces and overcoming the limitations of rigid substrates in complex practical scenarios.

**Figure 5:**
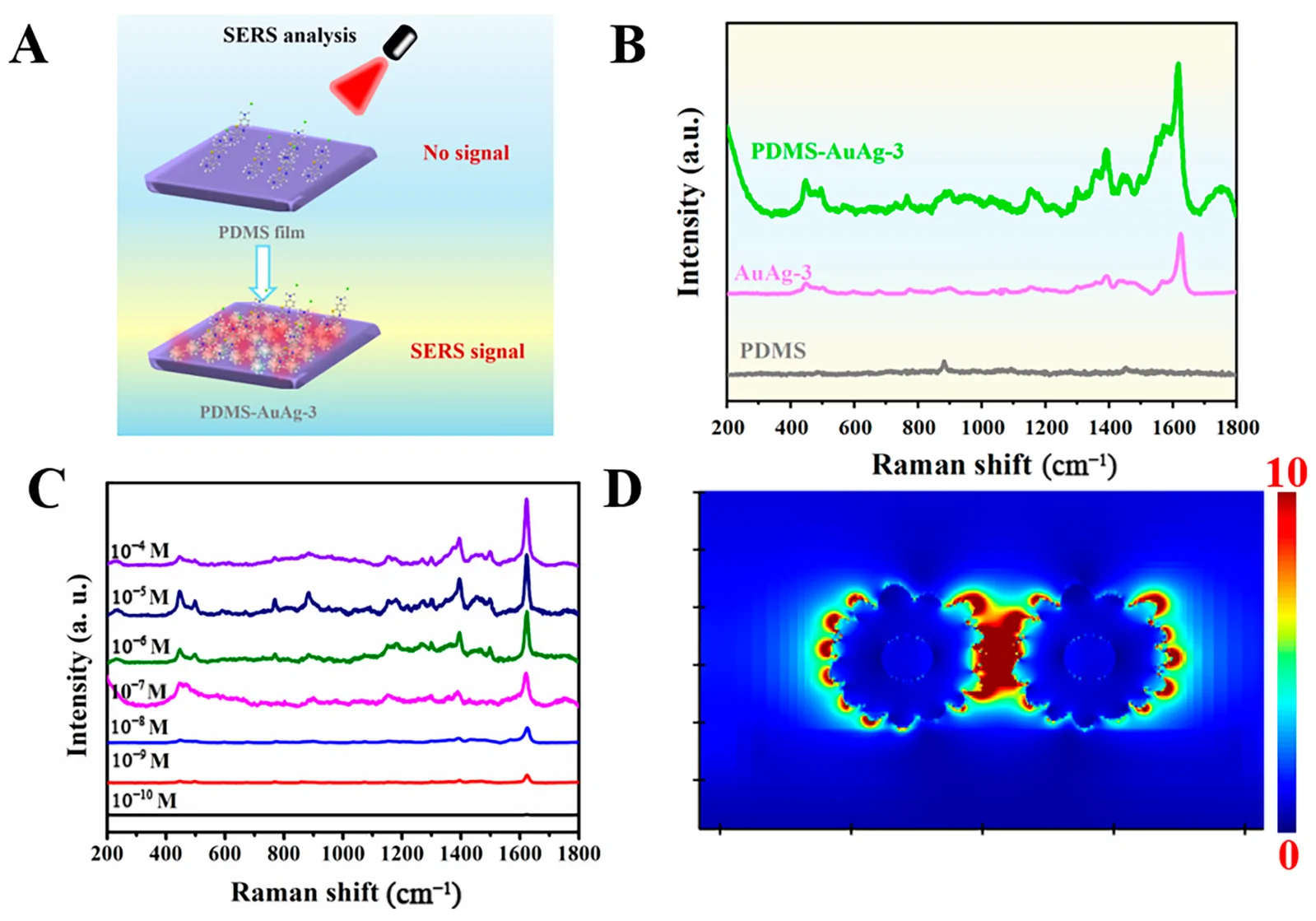
(**A**) Schematic diagram of flexible SERS substrate with SERS signal enhancement performance; (**B**) SERS spectra of MB (10^−5^ M) adsorbed on PDMS, AuAg-3 and PDMS-AuAg-3; (**C**) SERS spectra for MB at concentrations ranging from 10^−4^ to 10^−10^ M; (**D**) simulated electric field distribution of PDMS-AuAg-3.

Subsequently, SERS performance of PDMS-AuAg-3 substrate was evaluated using MB as the Raman reporter (Figure 5A). As revealed in Figure 5B, PDMS-AuAg-3 exhibits significantly stronger signal amplification compared to PDMS and AuAg-3. Figure 5C presents the SERS spectra of MB molecules at different concentrations ranging from 10^−4^ to 10^−10^ M. Although the corresponding SERS signal intensity decreases with the decrease in MB concentration, distinct SERS signals are detectable even at 10^−9^ M, indicating that the minimum experimentally tested concentration reaches 10^−9^ M. For each concentration, five independent measurements (n = 5) were performed at random positions on the substrate. Figure S4 shows a strong linear correlation between the logarithm of MB concentration and SERS intensity at 1626 cm^−1^ (y = 1807.06x + 18,104.08, R^2^ = 0.974), indicating that our method has a good ability to quantitatively detect MB molecules over a wide range. In addition, the limit of detection (LOD) was calculated and the LOD value for MB was determined to be 2.4 × 10^−10^ M, which was based on blank measurements and the calibration slope. This exceptional sensitivity is attributed to the hydrophobic nature of the substrate, which prevents the spread of the analyte solution. By suppressing the coffee-ring effect, the substrate concentrates the analyte molecules into a smaller area during drying, thereby increasing the local molecular density and improving signal uniformity [48,49]. Additionally, FDTD simulations (Figure 5D) confirm that the PDMS-AuAg-3 flexible substrate maintains superior electric field enhancement compared to dispersed AuAg-3 nanostars. In summary, the PDMS-AuAg-3 substrate prepared by the three-phase interface transfer process effectively overcomes the signal heterogeneity issues associated with conventional liquid-phase SERS substrates. By combining the intrinsic electromagnetic enhancement of the hollow nanostars with the hydrophobic surface-induced analyte enrichment, this flexible platform achieves high sensitivity, good signal uniformity, and broad applicability, providing a robust foundation for rapid on-site screening of trace substances.

## 3. Materials and Methods

### 3.1. Materials and Instruments

Silver nitrate (AgNO_3_), gold (III) chloride trihydrate (HAuCl_4_·3H_2_O) and ethanol were purchased from Aladdin Reagent Co., Ltd. (Shanghai, China). Hydroquinone (98%). Sodium citrate dihydrate (Na_3_C_6_H_5_O_7_·2H_2_O) and poly(vinylpyrrolidone) (PVP, M.W.: 250,000) were obtained from Shanghai Macklin Biochemical Co., Ltd. (Shanghai, China). MB, dichloromethane (CH_2_Cl_2_), and hexane were purchased from Sigma-Aldrich Shanghai Trading Co., Ltd. (Shanghai, China). PDMS Sylgard 184 elastomer was obtained from Dow Corning Co., Ltd. (Midland, MI, USA). All chemical reagents were analytical grade and used without further purification.

The morphology of the prepared samples was characterized using a transmission electron microscope ((TEM; Talos F200X G2, Thermo Fisher Scientific, Waltham, MA, USA). The ultraviolet–visible (UV–Vis) spectra were recorded using a UV 3600 spectrophotometer (Shimadzu Corporation, Kyoto, Japan). SERS spectra were acquired using a LabRAM Odyssey Raman spectrometer (HORIBA France SAS, Longjumeau, France) equipped with a 633 nm excitation laser.

### 3.2. Preparation of AuAg Alloy Nanostars

**Scheme 1:**
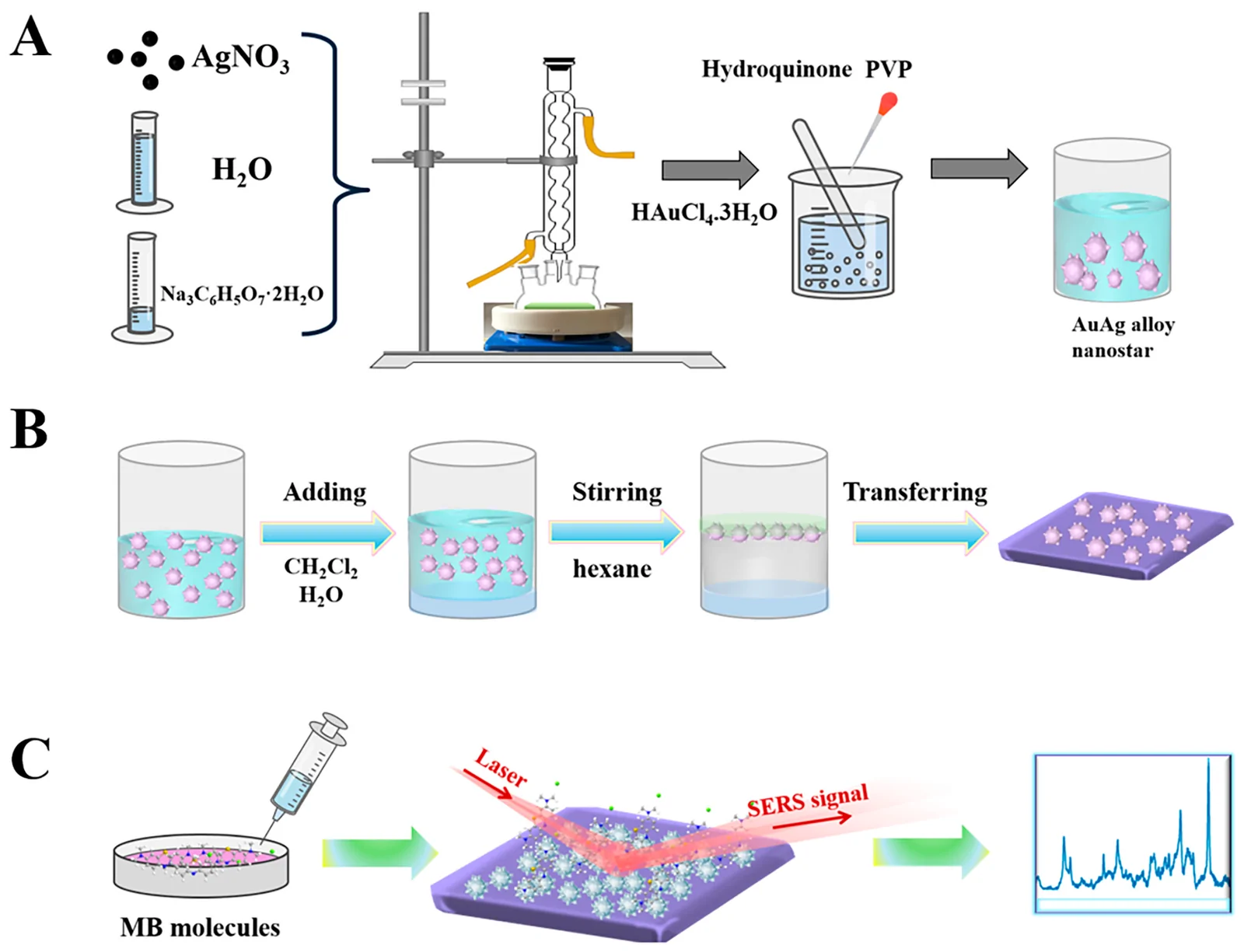
Schematic diagram of the preparation process of (**A**) AuAg alloy nanostars and (**B**) PDMS-AuAg alloy nanostar films, as well as (**C**) the overall workflow of SERS detection.

As shown in Scheme 1A, AuAg alloy nanostars were synthesized via a two-step process. The first step was to prepare Ag seeds using a citric acid reduction method. Firstly, 40 mg of AgNO_3_ powder was dissolved in 200 mL of deionized water and heated to 100 °C for 25 min. Then, 20 mg of Na_3_C_6_H_5_O_7_·2H_2_O was accurately weighed using an analytical balance and immediately added to the boiling solution. The mixture was maintained at boiling temperature for 50 min until the color of the solution turned yellow-green, indicating the formation of Ag seeds.

The second step was to prepare AuAg alloy nanostars using Ag seeds as templates at room temperature. A total of 150 μL of HAuCl_4_·3H_2_O aqueous solution (24.3 mm) and 1 mL of Ag seed solution were sequentially added to 10 mL of deionized water under continuous stirring. The color of the solution could be observed to change from light yellow to light blue. After stirring for 30 min, 10 mg of PVP (accurately weighed) and 1 mL of hydroquinone aqueous solution (33 mm) were added, and the mixture was stirred for an additional 30 min to facilitate the growth of AuAg alloy nanostars, resulting in a deep blue solution. In order to modulate the LSPR properties of AuAg alloy nanostars, the addition volume of Ag seed solution was varied (1, 2, 3 and 4 mL), yielding samples designated as AuAg-1, AuAg-2, AuAg-3 and AuAg-4, respectively. After synthesis, all AuAg alloy nanostar samples were centrifuged at 8500 rpm for 20 min, washed with deionized water, and redispersed in the same volume (1 mL) of deionized water to ensure comparable concentrations.

### 3.3. Preparation of PDMS Films and PDMS-AuAg Alloy Nanostar Films

To prepare the flexible PDMS substrate, 2.0 g of Sylgard 184 silicone elastomer base and 0.2 g of curing agent were thoroughly stirred in a beaker using the plastic rod. The mixture was poured into a mold and placed in a vacuum drying oven to degas. Following the removal of air bubbles, the mixture was cured at 60°C for 2 h to obtain a smooth PDMS film, which was subsequently cut into 0.5 × 0.5 cm^2^. Finally, PDMS films were treated with UV–ozone radiation to activate the surface from hydrophobic to hydrophilic for further use, which is a prerequisite to ensure the successful and uniform transfer of the AuAg alloy nanostar film via the three-phase interfacial method.

A three-phase interfacial self-assembly method was used to fabricate PDMS-AuAg alloy nanostar films, as depicted in Scheme 1B. Specifically, 0.3 mL of AuAg alloy nanostars solution, 0.2 mL of ethanol, 2 mL of CH_2_Cl_2_, and 2 mL of deionized water were sequentially added to a centrifuge tube and allowed to equilibrate for 2 min to stabilize the interfaces. Subsequently, the mixed solution was vigorously shaken and allowed to stand for 20 s, and a light-blue thin film could be observed. Then, 1 mL of hexane was added down the tube wall. Finally, the top layer of hexane and water phase were removed carefully, and then PDMS was moved inside under the liquid to transfer the AuAg alloy nanostar film, ultimately forming PDMS-AuAg alloy nanostar films. In summary, flexible PDMS-AuAg alloy nanostar films were prepared using a UV–-ozone assisted interface self-assembly strategy. The temporary hydrophilic modification of PDMS ensures the integrity of the nanostar monolayer, while the inherent hydrophobicity of the PDMS matrix ultimately drives effective analyte enrichment through solvent evaporation-induced repulsion effects.

### 3.4. SERS Detection Process

The protocol for SERS detection of MB molecules is illustrated in Scheme 1C. Firstly, MB powder was dissolved in deionized water to prepare stock solutions with concentrations ranging from 10^−4^ to 10^−10^ M, which were stored in the dark for future use. Subsequently, the prepared PDMS-AuAg alloy nanostar substrates were incubated in 1 mL of MB solutions at different concentrations for 30 min, followed by drying at room temperature for 1 h. Finally, the MB-loaded substrates were placed on a glass slide for SERS spectral acquisition. SERS measurements were performed equipped with a 633 nm excitation laser. The laser power was set to 17 mW with 50% attenuation, and the acquisition time was 10 s per spectrum.

### 3.5. FDTD Calculations

FDTD simulations were performed using Lumerical FDTD 2020 Solutions software to investigate the electromagnetic field distribution of adjacent AuAg alloy nanostars (AuAg-1, AuAg-2, AuAg-3 and AuAg-4). The geometric parameters used in the simulations were derived from the TEM images of the synthesized samples. A plane wave with a wavelength of 633 nm, polarized along the x-direction, was used as the excitation source, incident from the top. Perfectly matched layers (PML) were applied in all directions (x, y, z) as boundary conditions to absorb outgoing waves. To achieve high resolution, the total simulation time and mesh size were set to 1000 fs and 1 nm, respectively.

## 4. Conclusions

In summary, we have successfully developed a flexible SERS platform by integrating morphology-tuned hollow AuAg alloy nanostars with a hydrophobic PDMS substrate. By precisely varying the Ag seed concentration, the morphology of the nanostars was systematically tailored from solid structures with sparse branches to hollow structures with tunable branch characteristics. FDTD simulations and experimental results identified the hollow AuAg-3 nanostars as the optimal SERS substrate due to intense electromagnetic field enhancement arising from the synergistic effect of tip-enhanced plasmonics. Furthermore, interfacial self-assembly of AuAg-3 nanostars onto flexible PDMS yielded a hydrophobic substrate that effectively enriches analyte molecules during solvent evaporation and suppresses the coffee-ring effect, thereby enhancing signal uniformity and sensitivity, enabling the detection of MB at concentrations down to 2.4 × 10^−10^ M. This work provides valuable insights into the design of bimetallic nanostructures and establishes a robust strategy for fabricating flexible, sensitive SERS sensors that are suitable for future real-world applications.

## Figures and Tables

**Table 1:** EDS-determined weight and atomic percentages of Au and Ag in AuAg-1, AuAg-2, AuAg-3 and AuAg-4.

Sample	Au (wt.%)	Ag (wt.%)	Au (at.%)	Ag (at.%)
AuAg-1	99.24	0.76	98.63	1.37
AuAg-2	96.28	3.72	93.41	6.59
AuAg-3	92.89	7.11	87.74	12.26
AuAg-4	90.59	9.41	84.06	15.94

## Data Availability

The original contributions presented in this study are included in the article/Supplementary Material. Further inquiries can be directed to the corresponding author(s).

## Supplementary Materials

The following supporting information can be downloaded at https://www.mdpi.com/article/10.3390/molecules31193561/s1: Figure S1: TEM image of Ag seeds; Figure S2: Weight and atomic percentage of Au and Ag of AuAg-1 (a), AuAg-2 (b), AuAg-3 (c) and AuAg-4 (d) obtained using EDS analysis; Figure S3: Raman spectrum of pure MB molecules; Figure S4: Linear correlation between logarithm of MB concentration and SERS intensity at 1626 cm^−1^.

## References

[1] Chen J., Yang J., Lu Y., Dai Z., Li H. (2026). Dual-signal amplified SERS sensing based on aptamer-modified Au/Fe_3_O_4_ nanohybrids and Ag NPs-functionalized bacterial cellulose film for on-site marine toxin analysis. Sens. Actuators B Chem..

[2] Gu Z., Chen T., Zu D., Yang A., Huang W., Wu Y., Gan Y., Chen H., Yang J., Yu X. (2025). Versatile spiky spindle-shaped copper-based nanocomposites: A SERS substrate for discrimination, quantification and inactivation of multiple bacteria. J. Colloid Interface Sci..

[3] Liu S., Ding Q., Zhao L., Bai Y., Li M., Lee J.-H., Zhu Y., Ding Y., Sun L., Liu Y. (2026). SERS signal unification strategy: Preparation of AuNPs@4-MBA and its quantitative detection in mixed lactic acid bacteria. Spectrochim. Acta Part A Mol. Biomol. Spectrosc..

[4] Guo H., Qian K., Cai A., Tang J., Liu J. (2019). Ordered gold nanoparticle arrays on the tip of silver wrinkled structures for single molecule detection. Sens. Actuators B Chem..

[5] Guo X., Cai X., Lei F., Jiao Y., Zhao X., Li Z., Zhang C., Man B., Yu J. (2024). Both-In-One Rapid Detection and Removal of Methylmercury in Real Complex Aquatic Environments Using a Janus Confined SERS Filter Membrane. Adv. Funct. Mater..

[6] Yang Z., Yang L., Liu Y., Chen L. (2024). Photocatalytic Deposition of Au Nanoparticles on Ti_3_C_2_Tx MXene Substrates for Surface-Enhanced Raman Scattering. Molecules.

[7] Zhong Y., Li J., Xu P., Lou Y., Zhang X., Ji H., Teng Y. (2026). Liquid-liquid interfacial self-assembly of ordered CuS@cu core-shell heterostructure for synergistic SERS enhancement. Spectrochim. Acta Part A Mol. Biomol. Spectrosc..

[8] Gokulakrishnan J., Koppole K. (2024). Green synthesized Ag/rGO embedded flexible cotton SERS substrate for methylene blue detection. Opt. Mater..

[9] Kan L., Zhao W., Liu M., Pan W., Li D., Zhao Y., Jiang L. (2025). Ratiometric SERS sensor for sensitive quantification of methicillin-resistant Staphylococcus aureus using Ti_3_C_2_@AuNP films and aptamer-based tags. Appl. Surf. Sci..

[10] Ma X., Fu L., Zhang L., Bao M., Zhou J., Wang H., Zhang Q., Xia J. (2026). A multifunctional MoS_2_/Au/FP flexible SERS substrate: Construction with triple integration of signal amplification, self-calibration stability and photocatalytic self-cleaning. Spectrochim. Acta Part A Mol. Biomol. Spectrosc..

[11] Kadavath J.K., Pelaes R.F.C., Guzman S.S., Gomez N.A.G., Avellaneda D.A., Krishnan B., Shaji S. (2025). Selectively engineered plasmonic nanostructures by laser surface patterning for ultrasensitive SERS sensors. Microchem. J..

[12] Liu L., Zhao Q., Yin N., Xue Z., Sun Y., Wu Y. (2025). Self-assembled bimetallic Au@Ag nanostars monolayer flexible substrates for ultrasensitive SERS. J. Alloys Compd..

[13] Zhou N., Zeng Y., Huang Q., Tan R., Chen Y., Zhong L., Luo X., Deng X. (2025). A SERS aptasensor based on Au@Ag bimetallic nanostars-magnetic covalent organic composites for rHuEPO-α detection. Anal. Chim. Acta.

[14] Gao X., Hou X., Ma Z., Xiao C., Jia L. (2025). Highly dispersed Ag nanocrystals functionalized ZIF-8 derived ZnO hollow structures for superior sensitive and selective detection of nitric oxide. Sens. Actuators B Chem..

[15] Feng Z., Jia Y., Cui H. (2024). Engineering the surface roughness of the gold nanoparticles for the modulation of LSPR and SERS. J. Colloid Interface Sci..

[16] Zhao W., Yang S., Zhang D., Zhou T., Huang J., Gao M., Zhang X., Liu Y., Yang J. (2023). Multi-dimensional plasmonic coupling system for efficient enrichment and ultrasensitive label-free SERS detection of bilirubin based on graphene oxide-Au nanostars and Au@Ag nanoparticles. J. Colloid Interface Sci..

[17] Ma J., Liu X., Wang R., Zhang J., Jiang P., Wang Y., Tu G. (2020). Bimetallic Core–Shell Nanostars with Tunable Surface Plasmon Resonance for Surface-Enhanced Raman Scattering. ACS Appl. Nano Mater..

[18] Cho H.-S., Yang C.-H., Kim Y.-H., Kim Y.J., Yoo K., Shin M., Bae H.-J., Jun B.-H. (2025). Silica nanoparticles surface-decorated with Au-Ag alloy nanoparticles as highly sensitive, reproducible, and stable surface-enhanced Raman scattering (SERS) substrates. J. Alloys Compd..

[19] Tran N.L.N., Phuong N.T.T., Ta H.K.T., Van Hoang D., Van T.T., Van Le H., Tran N.H.T. (2025). Designing flexible SERS platforms by embedding double-plasmonic (Ag-Au) nanostructures in porous ZIF-8 frameworks on PDMS. Sens. Actuators A Phys..

[20] Lan W., Liu M., Mao L., Li Q., Cao Q.-E., Liu C., Zhou C.-H. (2025). A 2D nanoarray of core-shell AuNSs@4-MBA@Ag@Au NPs SERS substrate combined with ratiometric analysis for detection of thiram. Microchem. J..

[21] Mariappan P.R., Barveen N.R., Chiu C.-C., Kuo C.-Y., Cheng Y.-W. (2025). Dual enhancement of polyaniline-modified Au-Ag bimetallic nanostars as a flexible SERS substrate for real-time detection. Surf. Interfaces.

[22] Ren S., Fu J., Liu G., Zhang H., Wang B., Yu J. (2025). Ultrasensitive detection of methylene blue by surface-enhanced Raman scattering (SERS) with Ag nanoparticle-decorated magnetic CoNi layered double hydroxides. Anal. Methods.

[23] Wang H., Wu Y., Song H. (2019). Synergistic effects of photonic crystal and gold nanostars for quantitative SERS detection of 3-Phenoxybenzoic acid. Appl. Surf. Sci..

[24] Bian J., Liu M., Liu X., Bian X., Gu C., Ma J., Jiang T. (2025). SERS detection of pelvic infection-related pathogenic bacteria based on flexible PDMS-MXene@MOF@Ag ternary substrate. Microchem. J..

[25] Qian S., Zhao W., Guo R., Wang X., Dai H., Lang J., Kadasala N.R., Jiang Y., Liu Y. (2024). Apt-Conjugated PDMS-ZnO/Ag-Based Multifunctional Integrated Superhydrophobic Biosensor with High SERS Activity and Photocatalytic Sterilization Performance. Int. J. Mol. Sci..

[26] Chen J., Liu X., Tang J., Jiang J., Gu C., Jiang T., Wu K. (2025). Quantitative detection and intelligent distinguishing of urinary tract infection pathogens based on SERS-active PDMS@BP-CNT ternary substrate. Sens. Actuators B Chem..

[27] Niu F., Jiang P., Wang L., Gao Y., Zhu H., Gao N., Cai Z., He H., He Y., Chang G. (2024). Controllable growth of Au NBPs@Ag nanorods modified PDMS as SERS substrate for rapid detection of malachite green in aquatic products. Microchem. J..

[28] Wang M., Diao H., Dou C., Wu Z., Ruan L., Wang Z., Wang Z., Ye W., Duan J., Li Y. (2024). Label-free detection of pathogenic microorganism using Ag NPs@PDMS sponge SERS substrate and machine learning. Sens. Actuators B Chem..

[29] Zhou Y., Zhang W., Cheng S., Xing Y., Wang J., Yu F., Wang R. (2025). Flexible PDMS-SERS platform for culture-free diagnosis of bacterial infections in clinical wound care. Talanta.

[30] Guo Z., Zheng Y., Yin L., Xue S., Ma L., Zhou R., El-Seedi H.R., Zhang Y., Yosri N., Jayan H. (2024). Flexible Au@AgNRs/MAA/PDMS-based SERS sensor coupled with intelligent algorithms for in-situ detection of thiram on apple. Sens. Actuators B Chem..

[31] Zhang W., Luo K., Chen W., Chen M., Cai N., Li J. (2026). Highly SERS active composite of silver nanospheres-decorated Ag nanostar/ZIF-8 for determination of diquat and thiram residues. Spectrochim. Acta Part A Mol. Biomol. Spectrosc..

[32] Yang Y., Wang X., Hu Y., Liu Z., Ma X., Feng F., Zheng F., Guo X., Liu W., Liao W. (2025). Rapid enrichment and SERS differentiation of various bacteria in skin interstitial fluid by 4-MPBA-AuNPs-functionalized hydrogel microneedles. J. Pharm. Anal..

[33] Li J., Wang W., Zhao L., Rong L., Lan S., Sun H., Zhang H., Yang B. (2015). Hydroquinone-Assisted Synthesis of Branched Au–Ag Nanoparticles with Polydopamine Coating as Highly Efficient Photothermal Agents. ACS Appl. Mater. Interfaces.

[34] Wang H., Wang D., Huangfu H., Lv H., Qin Q., Ren S., Zhang Y., Wang L., Zhou Y. (2022). Branched AuAg nanoparticles coated by metal–phenolic networks for treating bacteria-induced periodontitis via photothermal antibacterial and immunotherapy. Mater. Des..

[35] Wang H., Fang T., Liu H., Wei T., Dai Z. (2022). Gold Nanostar-Based Sensitive Catechol Plasmonic Colorimetric Sensing Platform with Ultra-Wide Detection Range. Chemosensors.

[36] Yang W., Geng W., Lu X., Qian L., Luo S., Xu L., Shi Y., Song T., Li M. (2025). Unveiling the Photocatalytic Behavior of PNTP on Au-Ag Alloy Nanoshells Through SERS. Catalysts.

[37] Li Y.-L., Zhu J., Weng G.-J., Liu Y.-N., Li J.-J., Zhao J.-W. (2023). Study on the roughen process of branches of AuAg nanostars for the improved surface-enhanced Raman scattering (SERS) to detect crystal violet in fish. Sens. Actuators B Chem..

[38] Zhang G., Hu H., Deng S., Xiao X., Xiong Y., Peng J., Lai W. (2023). An integrated colorimetric and photothermal lateral flow immunoassay based on bimetallic Ag–Au urchin-like hollow structures for the sensitive detection of *E. coli* O157:H7. Biosens. Bioelectron..

[39] Ghosal M., Mondal S., Ghosh T., Prusty D., Senapati D. (2025). Core-to-Shell Thickness-Regulated Ag@Au Nanocatalyst for LSPR-Improved In Situ Detection of Extracellular Peroxide: Response in a Cancer Cell. Anal. Chem..

[40] Han Q., Zhang H., Kaplan A., Fan S., Xu Y., Zhang C., Gao W., He E., Dong J. (2026). Synthesis of flexible SERS sensing platform of graphene/Ag-popcorns/PMMA for ultrasensitive detection. Spectrochim. Acta Part A Mol. Biomol. Spectrosc..

[41] Wang Y., Wang S., Zhao J., Liu Y., Yang H., Ruan W., Zhao B. (2025). Developing a Tunable Synthesis Route for Hollow Gold Nanoparticles@Semiconductor Core–Shell Heterostructures with Controllable Localized Surface Plasmon Resonance. Inorg. Chem..

[42] Chen B., Fan L., Li C., Xia L., Wang K., Wang J., Pang D., Zhu Z., Ma P. (2024). Au nanoparticles decorated β-Bi_2_O_3_ as highly-sensitive SERS substrate for detection of methylene blue and methyl orange. Analyst.

[43] Siegel A.L., Baker G.A. (2021). Bespoke nanostars: Synthetic strategies, tactics, and uses of tailored branched gold nanoparticles. Nanoscale Adv..

[44] Wang X., Chang S., Zhang X., Guo X., Xu Y., Yang D., Luo L., Wang J., Wu H., Cui Y. (2024). A highly sensitive dual-mode lateral flow immunoassay based on plasmonic hollow Ag/Au nanostars enhancing light absorption. Talanta.

[45] Schiavi S., Taglietti A., Magni A., Galinetto P., Albini B. (2025). Increasing SERS performance of silver nanoparticles with nanometric coating of polydopamine: A novel approach for methylene blue detection. Appl. Surf. Sci..

[46] Chen L., Zhao X., Yang L., Guo S., Park E., Zhao B., Han X.X., Jung Y.M. (2025). Three-dimensional NTF–Ag composites for ultrasensitive SERS-based detection of malachite green on crucian carp skin. Spectrochim. Acta Part A Mol. Biomol. Spectrosc..

[47] Song X., Zhang Y., Ren X., Tang D., Zhang X., Li X. (2024). Self-cleaning SERS sensor based on flexible Ni_3_S_2_/MoS_2_@Ag@PDMS composites for label-free multiplex volatile organic compounds detection. Nano Res..

[48] Feng Y., Xia J., Zhang H., Han L., Du X., Wang J., Tian M. (2025). Flexible and reusable nanobowl-based Ag NPs@Ag-PDMS substrate for enhanced SERS detection. Microchem. J..

[49] Ding W., Xia Y., Song H., Li T., Yang D., Dong A. (2024). Macroscopic Superlattice Membranes Self-Assembled from Gold Nanobipyramids with Precisely Tunable Tip Arrangements for SERS. Angew. Chem. Int. Ed..

