# Supplementary material for: Interfacially Assembled Hollow AuAg Nanostars on Flexible PDMS for Sensitive SERS Detection
Source: Molecules. 2026 Oct 7;31(19):3561. doi: 10.3390/molecules31193561 (PMC13666804; doi:10.3390/molecules31193561)
Supplement: Supplementary file 1 [file molecules-31-03561-s001.zip › molecules-4542153-supplementary.pdf]

Interfacially Assembled Hollow AuAg Nanostars on Flexible PDMS for Sensitive SERS Detection

Jiatong Li [1], Meiyu Liu [1], Xinyuan Meng [2], Jiayi Hou [1], Chaojie Xu [1], Wenshi Zhao [2,*] and Jie Zhao [1,*]

[1] Key Laboratory of Bionic Engineering, Ministry of Education, Jilin University, Changchun 130022, China; lijt1824@mails.jlu.edu.cn (J.L.); liumeiyu1234562025@163.com (M.L.); jyhou25@mails.jlu.edu.cn (J.H.); xucj25@mails.jlu.edu.con (C.X.)
[2] Key Laboratory of Functional Materials Physics and Chemistry of the Ministry of Education, Jilin Normal University, Changchun 130103, China; 18943031115@163.com

Keywords:

surface-enhanced Raman scattering; PDMS-AuAg alloy nanostars; finite-difference time - domain; methylene blue

---

* Corresponding authors.

E-mail: 2405006@jlnu.edu.cn (W.S. Zhao), jiezhao@jlu.edu.cn (J. Zhao)

ABSTRACT

Optimizing the morphology and structure of noble metal nanomaterials is critical for the design of high-performance surface-enhanced Raman scattering (SERS) substrates for trace analyte detection. A plasmon-tunable SERS platform consisting of AuAg alloy nanostars anchored on a hydrophobic polydimethylsiloxane (PDMS) matrix was developed, which combined the chemical stability of Au and the strong localized surface plasmon resonance of Ag. AuAg alloy nanostars with sharp tips were synthesized via a galvanic replacement reaction, in which the precise modulation of Ag seed concentration enabled a morphological evolution from solid to hollow nanostructures. The optimized AuAg-3 nanostars exhibited the highest SERS response due to a dual synergistic mechanism: strong electromagnetic enhancement at the sharp branched tips and additional plasmonic enhancement from the hollow cavity through light scattering and trapping, which increased the optical path length and excitation efficiency. Furthermore, these nanostars were transferred onto a flexible PDMS film through interfacial self-assembly. The resulting hydrophobic PDMS-AuAg-3 substrate synergistically combined the strong electromagnetic enhancement from the nanostars with efficient analyte enrichment driven by the hydrophobic surface, achieving an ultra-low detection limit of 2.4×10^{-10} M for methylene blue (MB). This strategy effectively balances high sensitivity and excellent re-producibility, offering a robust pathway for the development of next-generation flexible SERS sensors.

Figure S1

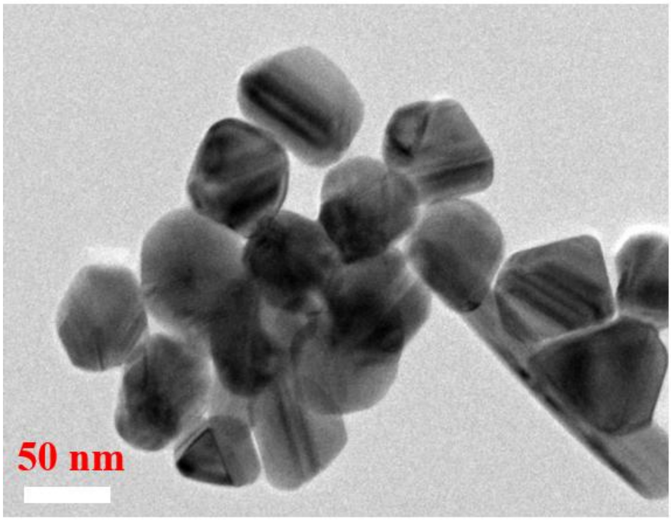

Figure S1. TEM image of Ag seeds.

Figure S2

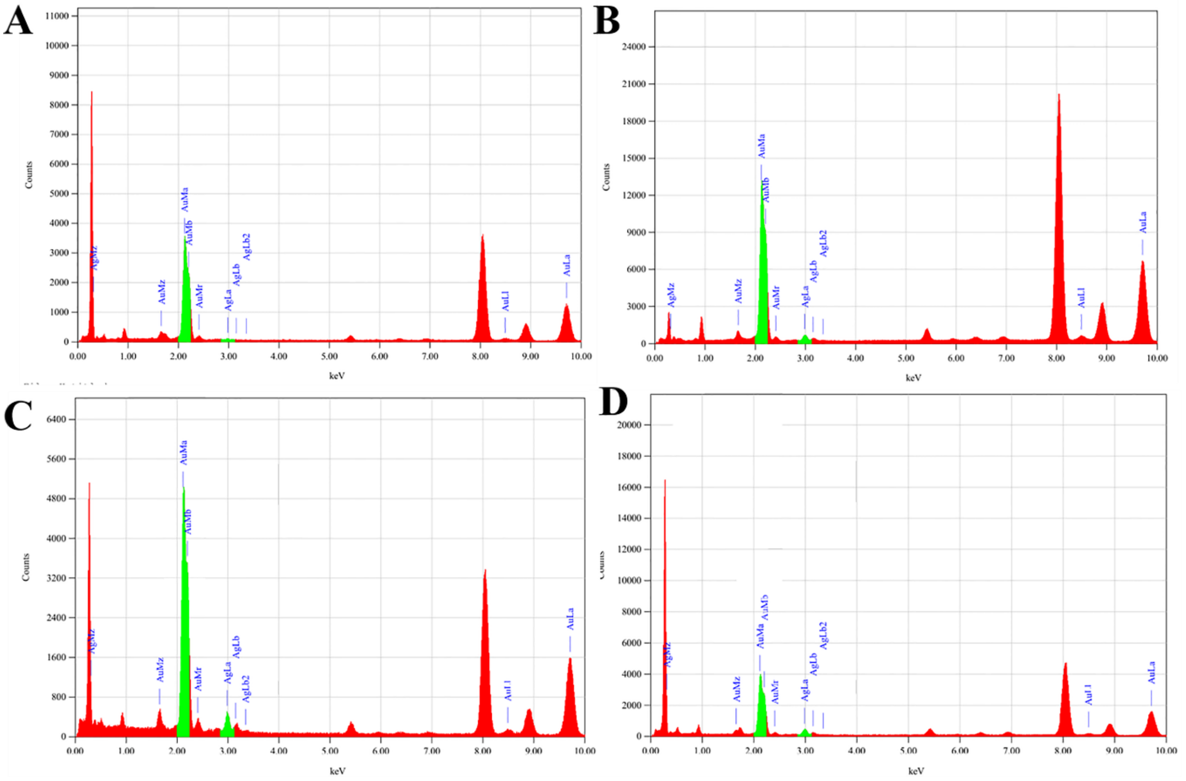

Figure S2. Weight and atomic percentage of Au and Ag of AuAg-1 (a), AuAg-2 (b), AuAg-3 (c) and AuAg-4 (d) obtained using EDS analysis.

Figure S3

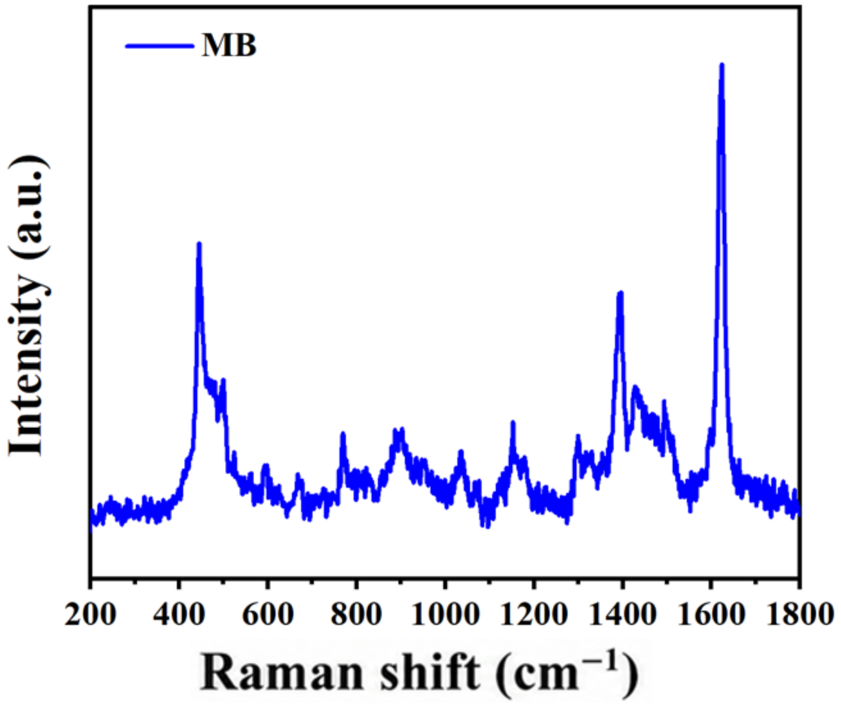

Figure S3. Raman spectrum of pure MB molecules.

Figure S4

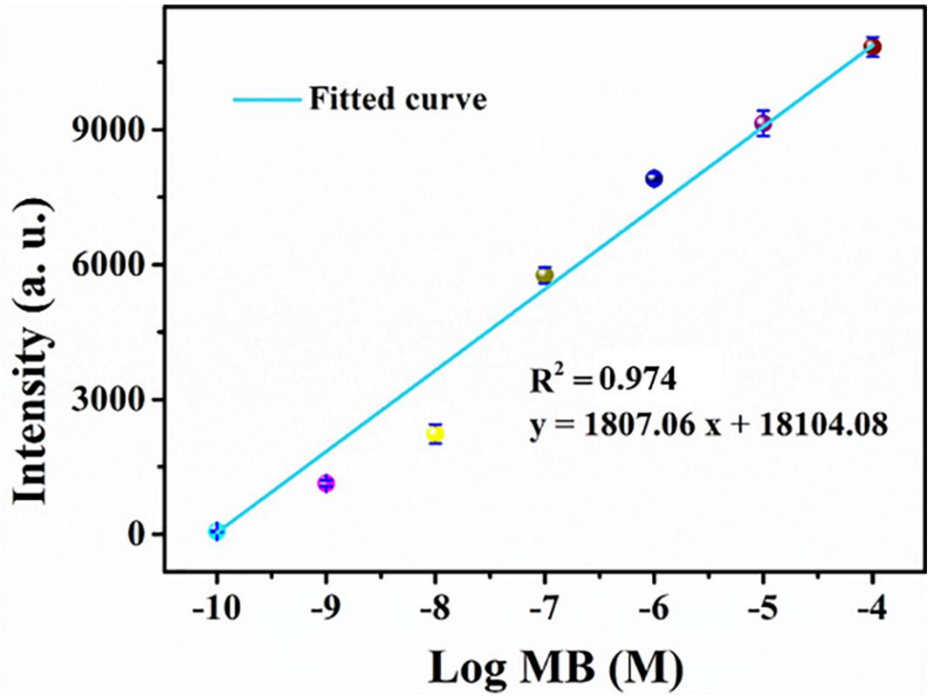

Figure S4. Linear correlation between logarithm of MB concentration and SERS intensity at 1626 cm^{-1}.
